# Metallothionein and Glutathione Content as Biomarkers of Metal Pollution in Mussels and Local Fishermen in Abu Qir Bay, Egypt

**DOI:** 10.5696/2156-9614-6-12.50

**Published:** 2017-02-21

**Authors:** Aziza A. Saad, Amany El-Sikaily, Hany Kassem

**Affiliations:** 1 Applied Medical Chemistry, Medical Research Institute, Alexandria University, Egypt; 2 Marine Pollution Department, National Institute of Oceanography and Fisheries, Ministry of Scientific Research, Egypt

**Keywords:** metallothionein, malondialdehyde, biomarker, metal pollution

## Abstract

**Background.:**

When heavy metals accumulate in air, soil, and water, the risk of human exposure increases among industrial workers, as well as in people living near polluted areas. Heavy metals adversely affect a variety of bodily systems such as the cardiovascular, respiratory, endocrine, immune, and reproductive systems. In addition, long-term exposure and accumulation of heavy metals in the body may disturb oxidative stress genes and thus increase the susceptibility to various diseases.

**Objectives.:**

The aim of this study is to estimate the metallothionein concentration in both mussel samples from Abu Qir Bay, Egypt and the blood of local fishermen as a biomarker of exposure to metal pollution.

**Methods.:**

Levels of metallothionein and heavy metals were measured in mussels. Blood levels of metallothionein and heavy metals of local fishermen were measured and compared with a control group. The effect of heavy metal exposure on oxidative stress status was investigated through the determination of malondialdehyde (MDA), catalase and glutathione content.

**Results.:**

The results of this study showed high concentrations of metallothionein in mussels and in fishermen's blood, accompanied by high concentrations of metals such as cadmium (Cd), copper (Cu), lead (Pb), chromium (Cr), and zinc (Zn). At the same time, a significant decrease in glutathione content and catalase enzyme activity was associated with a significant increase in the malondialdehyde concentrations in sera of fishermen.

**Conclusions.:**

The present study found that the El Maadiya region is polluted with heavy metals, inducing oxidative stress in fishermen in the vicinity. These results reveal the necessity of further environmental monitoring in the study area in order to evaluate other types of pollutants and their effects on human health.

## Introduction

Heavy metal contamination in marine ecosystems is of global concern.^1^ The level of contamination of the aquatic environment due to heavy metals can be estimated by analyzing water, sediments and marine organisms. The levels of heavy metals in mollusks and other invertebrates are often considerably higher than in other constituents of the marine environment due to their habitat and feeding habits.^2–5^

The most commonly used biomarker for pollution in the marine environment is metallothionein, which has been particularly useful as a monitoring device, namely as a contaminant-specific indicator of metal exposure.^6^

Metallothioneins are inducible proteins; heavy metal cations accumulated within the cells stimulate metalloproteinneosynthesis by enhancing metallothionein gene transcription. The metallothionein messenger ribonucleic acid is translated by the cytosolic free ribosome; it leads to an increase in apo-metallothionein that rapidly reacts with the free metal cations that are present in the cytosol.^7^ Due to their biochemical and functional characteristics, metallothioneins are able to protect cell structures from non-specific interactions with heavy metal cations and to detoxify the metal excess penetrating into the cell. Due to their inducibility to heavy metals, metallothioneins are usually considered to be important specific biomarkers that detect an organism's response to inorganic pollutants such as cadmium (Cd), mercury (Hg), copper (Cu), and zinc (Zn) that are present in the aquatic environment.

Bioindicator organisms that have been commonly employed in the application of metallothioneins as biomarkers are fish, mollusks and crustaceans. Metallothioneins as tools for biomonitoring activities are important as they are ubiquitary proteins and therefore can be studied in most living organisms.^7^

**Abbreviations**
*Cd*: Cadmium
*MDA*: Malondialdehyde
*Cr*: Chromium
*Pb*: Lead
*Cu*: Copper
*RO*: Reactive oxygen species
*DNA*: Deoxyribonucleic acid
*Zn*: Zinc
*GSH*: Glutathione

The potential of metals in generating reactive oxygen species (ROS) and thus altering cellular reduction-oxidation (redox) states is considered to be the most important mechanism involved in metal-induced carcinogenicity.^8^,^9^ Recent research suggests that chronic exposure to ROS causes oxidative stress by disrupting the balance between the levels of the produced ROS and the potential of cellular antioxidant systems to remove them. Persistent oxidative stress leads to changes in cellular redox homeostasis and abnormal activation of redox-sensitive signaling molecules. Oxidative stress also damages bio-macromolecules such as deoxyribonucleic acid (DNA), proteins, and lipids; it eventually induces a variety of chronic and degenerative diseases including cancer, cardiovascular disorders, diabetes, rheumatoid arthritis, and Alzheimer's and Parkinson's disease.^10^

The toxicity of heavy metals may be attributed to the binding of metals to sulfhydryl groups in proteins such as glutathione (GSH), resulting in an inhibition of activity, interference with structure, or displacement of an essential metal element leading to deficiency effects.^11^ Repairing stress-damaged proteins and chelation of metals involving heat shock proteins and metallothionein is thus recognized as a potential mechanism of metal detoxification. Although mechanisms by which heavy metals interact have not been clearly elucidated, a number of biomolecules, including GSH, metallothionein, and heat shock proteins have been predominantly recognized as major interactive mediators when evaluating interactions based on metal mixture exposure.^12^

The primary purpose of this study is to obtain quantitative estimations of metallothionein concentrations in mussels as biomarkers of exposure to heavy metals in order to monitor the pollution of Abu Qir Bay, Egypt (El Maadiya region). The second purpose of the study is to examine the impact of heavy metal pollution on residents of the study area through the determination of various metals in the blood of study subjects and the detection of metallothionein and oxidative stress through the estimation of malondialdehyde, blood glutathione and catalase enzyme activity.

## Methods

### Study Area

**Figure 1:**
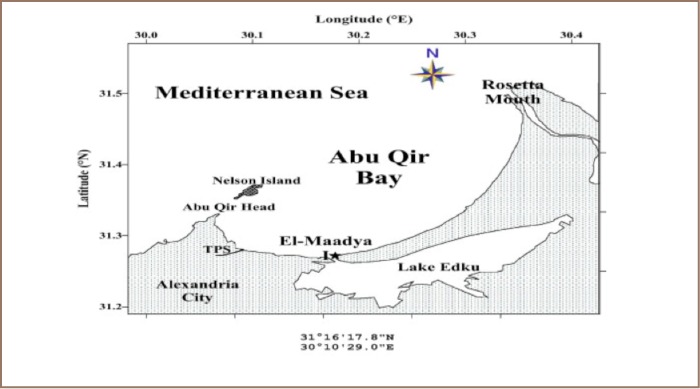
Map of Mediterranean Sea, Abu-Qir Bay showing the study area (El Maadiya region)

Abu Qir Bay is a shallow semicircular basin about 35 km east of Alexandria City between Abu Qir peninsula (west) and the Rosetta branch of the Nile (East), with a shoreline extending about 50 km (*Figure 1*).^13^ The bay is adjacent to one of the most populous, industrialized and commercialized coastal metropolitan areas in Egypt. The people of the region put the bay to a wide variety of recreational, commercial, and industrial uses. The bay contributes about 10% of the fish and shrimp catch from Egyptian Mediterranean waters. The bay is subjected to multiple pollution from two point sources: (i) Tabia pumping station; the station pumps to the bay (1.5–2.0) × 106 m^3^/day of industrial waste water get from 20 different factories, mostly textile, food processing and canning, mixed agricultural and domestic waste water and (ii) the outlet of Lake Edku.^13^

### Mussels

#### Mussel Sampling

Andaradulofii mussel samples were collected from the Mediterranean Sea, Abu-Qir Bay, in the El-Maadiya region and acclimated to laboratory conditions at 20°C for three days in ethylenediaminetetraacetic acid-free synthetic seawater, pH 7.9–8.0 and 35 osmolarity and salinity (Viarengo et al, 1997) to determine the presence of metallothionein and five other metals (cadmium, lead, chromium, copper and zinc).^14^

#### Mussel Analysis

Mussel metallothionein concentration was evaluated using a partially purified metalloprotein fraction which was obtained by acidic ethanol/chloroform fractionation of the tissue homogenate and measured colorimetrically using GSH (Viarengo A, et al, 1997).^14^ Mussel gills and digestive glands were rapidly dissected and then stored at 80°C for determination of metals content (Cd, Pb, Cr, Cu, and Zn) in mussels.^15^ All digested solutions were analyzed and measured using an atomic absorption spectrophotometer (SPECTR plus version) with an air-acetylene flame and deuterium background correction.

### Human Population

#### Sampling

A total of 56 male subjects, with an age range of 20–55 years old, and weight ranging from 62–85 kg were divided into two groups:

*Control (Group I)*: Included (12) healthy control male subjects living in the El-Maadiya region, but working in jobs other than fishing. They were free from diabetes, liver disease and thyroid dysfunction.

*Fishermen (Group II)*: Included (44) professional fishermen volunteers living in the El-Maadiya region. Information on age, weight, area of residence, smoking history, caffeine consumption, medication use, and history of acute or chronic illness were gathered via questionnaire. The food habits of study subjects were also taken into consideration. Subjects with thyroid dysfunction, diabetes, and liver disease were excluded.

Blood was collected from all subjects enrolled in this study for the assay of metallothionein, Cd, Pb, chromium Cr, and Zn, malondialdehyde (MDA), catalase and GSH content.

#### Ethical Approval

Written consent was provided by the study participants and approval was given by the ethics committee of Alexandra University (US Department of Health and Human Services, Registration of an Institutional Review Board, IORG0008812 Medical Research Institute, Expires 4/8/2019,OMB No: 0990-0279).

#### Analysis of Human Samples

Metallothionein concentration in human erythrocytes was evaluated using a partially purified metalloprotein fraction obtained by acidic ethanol/chloroform fractionation of erythrocytes hemolysate.^16^ The concentration of metallothionein is expressed as nanomoles of metallothionein per gram of hemoglobin (nmol MT/g Hb). Atomic absorption spectroscopy assay was used to determine the concentration of Cd, Pb, Cr, Cu, and Zn in blood samples of the study subjects.^17^ Blood metal content was obtained from a standard curve ranging from 0.25 to 5 mg/l.

Determination of serum lipid peroxide was based on the reaction of lipid peroxides with thiobarbituric acid in an acidic medium, which forms a red pigment that is extracted using n-butanol and measured at 530 nmol.^18^ The concentration of MDA is expressed as nmol MDA/ml.

Determination of blood glutathione content was based on the reduction of 5,5′ dithiobis- (2-nitrobenzoic acid) with glutathione to produce a yellow compound.^19^ The reduced chromogen was found to be directly proportional to the GSH concentration and its absorbance measured at 405 nm.

Determination of erythrocytes catalase activity was based on the conversion of hydrogen peroxide to water and oxygen.^20^ The apparatus was adjusted at zero using a blank cuvette. The reaction was started by adding hydrogen peroxide and was followed by a decrease in absorbance at intervals of 15 seconds and was measured at 240 nm.

### Quality Control

All the glassware and the Teflon cups were previously soaked overnight with 20% nitric acid and then rinsed. Each element concentration was estimated quantitatively according to the standard conditions described in the instrument manual. However, working standards of studied elements were prepared by diluting concentrated stock solutions with deionized water. Reagents of analytical grade were utilized for the blanks and calibration curves; precision was checked against a reference material (IAEA-433, International Atomic Energy Agency; Analytical Quality Control Services) which was analyzed with the digested mussel solutions. The measured concentrations of heavy metals were within the range of certified values with a recovery of 96.9–105.5%, and precision was found to be within 10%. The recoveries of studied metals for four replicates of the standard reference material were 105.5% for Cd; 96.9% for Cu; 105.2% for Cr; and 98.0% for Zn. The absorption wavelength and detection limits of heavy metals were as follows: 228.8 nm and 0.006 μg/g for Cd; 324.7 nm and 0.008 μg/g for Cu; 213.9 nm and 0.004 μg/g for Zn, respectively.

Absolute ethanol, chloroform, hydrochloric acid and nitric acid were of high performance liquid chromatography grade. Sodium citrate, 5,5′-dithiobis-2-nitrobenzoic acid, leupeptine, phenyl-methylsulphonylfloride, thiobarbituric acid, and reduced glutathione were purchased from Sigma-Aldrich Chemical Co., (St. Louis, MO, USA).

## Results

Table 1 depicts metallothionein concentrations in mussels collected from the El Maadiya region of the Mediterranean Sea. The results ranged from 1.37–32.6 μg/g wet weight. The concentrations of the five studied metals (Cd, Pb, Cr, Cu, and Zn) in mussels are also shown in Table 1.

**Table 1:** Metallothionein and Metal Concentrations in Mussels Collected from the El Maadiya Region of the Mediterranean Sea

	**MT**	**Cd**	**Pb**	**Cr**	**Cu**	**Zn**
**1**	2.53	1.11	50.32	10.58	1.77	9.97
**2**	1.37	0.72	47.48	8.78	1.23	2.48
**3**	32.6	0.57	45.86	9.07	0.75	6.28
**4**	1.77	0.81	15.42	11.14	0.75	3.06
**5**	1.37	0.86	16.23	11.05	1.07	1.53
**6**	1.37	0.78	15.42	9.16	0.80	5.04
**7**	19.15	0.72	13.80	10.95	1.71	3.74
**8**	12.11	0.72	13.80	9.54	1.39	6.02
**9**	11.1	0.75	18.67	10.10	1.39	3.62
**10**	23.81	1.00	12.99	9.54	1.28	3.08
**11**	8.69	0.95	19.48	8.69	0.64	5.20
**12**	7.73	1.13	20.29	13.31	1.28	3.20
**13**	1.71	0.80	118.49	8.88	1.28	2.31
**14**	3.97	0.59	15.83	9.07	1.39	3.99
	
**Mean**	9.23	0.822	30.29	9.99	1.195	4.25
**±SE**	1.94	0.046	7.69	0.347	0.094	0.576

Data represented as μg/g wet weight

Abbreviations: MT, Metallothionein; SE, standard error

Table 2 demonstrates the concentrations of metallothionein in the erythrocyte samples of the control and fishermen groups. Concentrations ranged from 6–30 nmol/gHb in the control group and from 11–151 nmol/gHb in fishermen. Differences between variables were measured by independent t-test and are shown in Table 3. Significantly higher levels of metallothionein were found in the fishermen group compared to the control group.

**Table 2:** Erythrocytes Metallothionein Concentration of Control and Fishermen Group

	**Control group (n = 12)**	**Fishermen group (n = 44)**	
	13	85	47
	8	22	19
	27	20	32
	22	77	23
	19	23	43
	6	37	40
	20	86	27
	18	37	23
	20	119	28
	30	25	11
	13	52	27
	9	151	16
		29	18
		143	42
		21	78
		20	20
		30	20
		37	24
		112	16
		50	16
		52	46
		73	23
	
**Mean**	17.1	44.1	
**±SE**	2.2	5.2	

Data expressed as nmol/gHb

Abbreviations: SE, standard error

**Table 3:** Statistical Analysis (Independent t-test)of Erythrocytes Metallothionein Concentrations of the Healthy Control and Fishermen Group

	**Group Statistics**		
	**Group 1**	**Group 2**	**Test of Significance**
**X̄**	17.1	44.1	P = 0.009 ^*^
**n**	12	44	
**Range**	(6 – 30)	(11 – 151)	
**SD**	7.5	34.2	
**±SE**	2.2	5.2	

Data expressed as nmol/gHb p< 0.05 indicates significance

Abbreviations: SD, standard deviation; SE, standard error

Table 4 illustrates the results of the blood concentration levels, in μg/ml, of the five studied metals (Cd, Pd, Cr, Cu and Zn) ranged from 0.052±0.029, 0.162±0.03, 0.199±0.025, 1.019±0.156, 0.063±0.016 and 0.157±0.018, 0.244±0.016, 0.244±0.009, 1.19±0.06,0.061±0.004 for both the control and fishermen groups, respectively.

**Table 4:** Whole Blood Concentrations of Cadmium (Cd), Lead (Pb), Chromium (Cr), Copper (Cu) and Zinc (Zn) in the Healthy Control and Fishermen Group

	**Control Group**					**Fisherman Group**				
	**Cd**	**Pb**	**Cr**	**Cu**	**Zn**	**Cd**	**Pb**	**Cr**	**Cu**	**Zn**
	ND	0.108	0.206	1.262	0.043	0.208	0.235	0.283	1.172	0.077
	0.088	0.127	0.254	0.958	0.087	0.256	0.372	0.303	1.733	0.102
	0.012	ND	0.110	1.291	0.014	0.316	0.500	0.146	–	0.022
	ND	0.186	0.109	–	0.019	0.022	0.225	0.116	–	0.019
	0.149	0.196	0.261	1.171	0.093	0.073	0.304	0.134	0.925	0.023
	0.009	0.284	0.255	1.059	0.131	0.184	0.314	0.201	–	0.024
	ND	0.225	0.263	1.265	0.097	0.184	0.147	0.246	1.326	0.091
	0.001	0.167	0.134	0.128	0.023	0.004	0.235	0.198	–	0.024
						0.018	0.314	0.301	1.056	0.035
						0.083	0.108	0.288	–	0.030
						0.020	0.304	0.243	1.385	0.101
						0.113	0.186	0.221	–	0.037
						ND	0.225	0.224	1.385	0.100
						ND	0.069	0.186	1.297	0.038
						ND	0.186	0.191	1.384	0.041
						0.019	0.392	0.225	1.064	0.101
						ND	0.049	0.194	0.879	0.052
						ND	0.196	0.233	1.567	0.052
						ND	0.176	0.203	0.841	0.053
						ND	0.147	0.221	0.868	0.053
						ND	0.206	0.264	1.064	0.052
						0.161	0.235	0.277	1.036	0.056
						0.247	0.265	0.270	–	0.064
						0.162	0.147	0.277	1.081	0.058
						0.291	0.147	0.297	–	0.063
						0.190	0.235	0.333	0.778	0.067
						0.305	0.157	–	1.413	0.087
						0.129	0.196	0.274	0.805	0.075
						0.228	0.167	0.310	–	0.078
						0.229	0.225	0.264	–	0.077
						0.132	0.294	0.282	1.557	0.080
						0.159	0.255	0.246	1.146	0.076
						0.238	0.225	0.288	1.794	0.083
						0.115	0.167	0.300	1.009	0.088
	
**Mean**	0.052	0.162	0.199	1.019	0.063	0.157	0.224	0.244	1.19	0.061
**±SE**	0.029	0.030	0.025	0.156	0.016	0.018	0.016	0.009	0.06	0.004

Data expressed as μg/ml.

Abbreviations: ND, not detected; SE, standard error

The serum malondialdehyde results of the healthy control and fishermen groups are shown in Table 5. They ranged from 0.8–2.6 nmol/ml and from 2.2–8.9 nmol/ml in the control and fishermen group, respectively. Statistical analysis using the independent t-test (*Table 6*) demonstrated significantly higher levels of malondialdehyde in the serum of the fishermen group compared to the control group.

**Table 5:** Serum Malondialdehyde Concentrations Across Study Groups

	**Control group**	**Fishermen group**	
	2.0	3.8	4.0
	2.3	3.3	3.4
	1.5	4.0	3.1
	2.4	3.0	3.0
	2.2	2.6	3.0
	2.1	2.8	3.6
	2.6	5.0	3.4
	1.5	2.3	2.2
	0.8	3.1	3.1
	1.3	2.7	3.3
		2.3	3.7
		8.9	3.4
		2.4	4.0
		3.6	4.0
		3.7	3.8
		4.9	3.8
		3.2	3.0
		6.1	2.5
		5.4	2.4
		4.5	2.2
	
**Mean**	1.87	44.1	
**±SE**	0.18	5.2	

Results presented as nmol/ml

Abbreviations: SE, standard error

**Table 6:** Statistical Analysis (Independent t-test) of Serum Malondialdehyde Concentrations of the Control and Fishermen Group

	**Group Statistics**		
	**Control group**	**Fishermen Group**	**Test of Significance**
**X̄**	1.87	3.56	P = 0.000 ^*^
**n**	10	40	
**Range**	(0.8 – 2.6)	(2.2 – 8.9)	
**SD**	0.57	1.23	
**±SE**	0.18	0.18	

Concentrations expressed as nmol/ml

^*^ p < 0.05 indicates significance

Abbreviations: SD, standard deviation; SE, standard error

Table 7 presents the blood glutathione content results, with mean concentrations of 39.6±1 and 24.4±0.6 mg/dL in the control and fishermen groups, respectively. Differences between the variables measured by independent t-test (*Table 8*) showed a significantly sharp decrease in blood glutathione levels in the fishermen group compared to the control group.

**Table 7:** Whole Blood Glutathione Content of the Control and Fishermen Group

	**Control group**	**Fishermen group**	
	35	29	21
	37	22	25
	35	26	27
	42	22	24
	40	29	22
	43	23	23
	41	32	28
	38	20	28
	41	23	28
	44	27	20
		25	22
		28	18
		25	19
		20	27
		25	30
		26	26
		20	32
		29	18
		24	18
		24	20
	
**Mean**	39.6	24.4	
**SD**	3.2	3.9	
**SE**	1.0	0.6	

Data expressed as mg/dL

Abbreviations: SD, standard deviation; SE, standard error

**Table 8:** Statistical Analysis (Independent t-test) of Glutathione Content in Whole Blood of the Control and Fishermen Group

	**Group Statistics**		
	**Control group**	**Fishermen Group**	**Test of Significance**
**X̄**	908	573.9	P = 0.000 ^*^
**N**	10	40	
**Range**	(805 – 991)	(308 – 702)	
**SD**	63.6	87.9	
**±SE**	20.1	13.9	

Data expressed asmg/dL

^*^ p < 0.05 indicates significance

Abbreviations: SD, standard deviation; SE, standard error

Data on erythrocyte catalase activity are presented in Table 9. Its concentration ranged from 805–991 units per gram of hemoglobin (U/gHb) and from 308–702 U/gHb for the control and fishermen groups, respectively. There was a very highly significant decrease in the enzymatic activity of erythrocyte catalase in the fishermen group compared to that of the control group (*Table 10*).

**Table 9:** Erythrocytes Catalase Enzymatic Activity Levels of the Control and the Fishermen Group

	**Control group**	**Fishermen group**	
	926	635	539
	991	550	633
	977	649	662
	985	690	510
	928	654	699
	843	456	662
	805	574	670
	874	670	517
	874	501	670
	877	443	556
		612	529
		648	526
		639	386
		563	509
		503	702
		618	646
		533	308
		569	499
		574	569
		528	554
	
**Mean**	908	573.9	
**±SE**	20.1	13.9	

Data expressed as U/gHb

Abbreviations: SE, standard error

**Table 10:** Statistical Analysis (Independent t-test) of Erythrocytes Catalase Enzymatic Activity Levels of the Control and Fishermen Group

	**Group Statistics**		
	**Control group**	**Fishermen Group**	**Test of Significance**
**X̄**	908	573.9	P = 0.000 ^*^
**n**	10	40	
**Range**	(805 – 991)	(308 – 702)	
**SD**	63.6	87.9	
**±SE**	20.1	13.9	

Data expressed as U/gHb

^*^ p < 0.05 indicates significance

Abbreviations: SD, standard deviation; SE, standard error

## Discussion

The results of this study indicate that the study area was contaminated with heavy metals such as cadmium, lead, copper, chromium, and zinc. These results are in agreement with previous studies which found that Abu Qir Bay is contaminated with heavy metals such as cadmium, nickel, cobalt, and aluminum.^2^,^5^,^21^ Our study demonstrates the presence of measurable amounts of metallothionein in mussel tissues collected from the study area.

Metallothioneins have been implicated in the homeostasis of essential metals such as Cu and Zn, and in the detoxification of excess levels of essential and nonessential metals in marine invertebrates.^22^ Several studies have demonstrated that metallothioneins protect organisms from metal toxicity due to their ability to bind metals like Cd, Cu, Zn and Hg. Metallothioneins also have the ability to increase their expression when metal concentrations in tissues rise.^23–27^

The findings of the study demonstrate that residents of the El Maadiya region face the immediate environmental impact of heavy metal pollution; however, no study in the available literature has addressed the effects of heavy metal contamination on the human population in this area.

The results of the current study demonstrate the presence of high concentrations of heavy metals such as cadmium, chromium, lead, copper and zinc in the blood of local fishermen. This has been linked to significantly high levels of metallothionein in the fishermen's erythrocytes. The most conspicuous feature of metallothionein is the inducibility of MT-1 and MT-2 genes by a variety of agents and conditions. The regulation of metallothionein biosynthesis occurs primarily at the level of transcription, where the cis-acting elements of DNA respond to transacting transcriptional regulatory proteins.

The MT-1 and MT-2 genes in higher species are rapidly induced in vitro and in vivo by a variety of stimuli including metals, hormones, cytokines, oxidants, stress and irradiation.^28^

Owing to their induction by a variety of stimuli, metallothioneins are considered to be valid biomarkers in the medical and environmental fields.^29^ Metallothionein is a remarkable protein, with a variety of cellular roles in homeostasis and the management of toxicant exposure.^30^ This small, cysteine-rich protein can combine with divalent heavy metal cations via cysteine associated thiols. Under normal circumstances, metallothionein is associated with zinc and copper cations, and thus serves as an important reservoir of these essential metals, donating them to apoenzymes and other apoproteins that require these metals to function.^31^ Other metals such as mercury and cadmium can also combine with metallothionein, which limits their toxic effects. Metallothionein's cysteine-associated thiols can also serve as anti-oxidant moieties that protect DNA and other macromolecules from the damaging effects of reactive oxygen species. The regulation of ROS levels can indirectly regulate nuclear factor kappa-light-chain-enhancer of activated B cells (NF-κB) activity, but metallothionein has also been found to directly interact with NF-κB and may regulate NF-κB-responsive genes via that direct interaction.^32^,^33^

**Figure 2:**
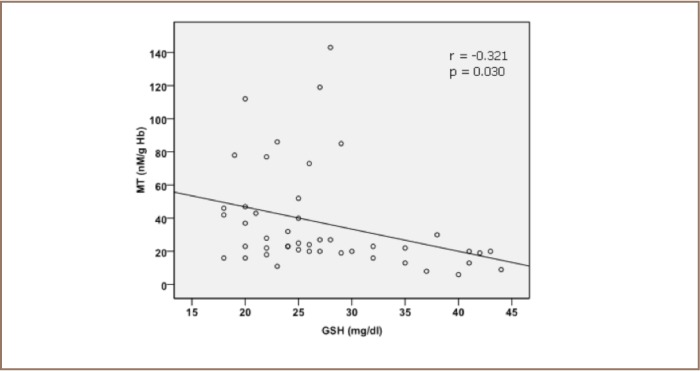
Pearson correlation between erythrocytes metallothionein concentration (MT) and blood glutathione content (GSH)

**Figure 3:**
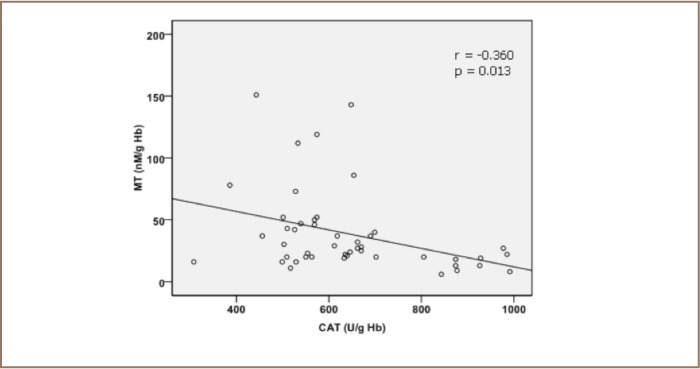
Pearson correlation between erythrocytes metallothionein concentration (MT) and erythrocytes catalase enzymatic activity

A hypothesis has been proposed that metallothionein acts as a chaperone during synthesis and modulation of metalloproteins and metallothionein and appears to be stabilized at high cellular GSH concentrations. Metal-requiring apoenzymes can abstract metals from metallothionein as demonstrated in vitro. Glutathione can form a complex with metallothionein. Subsequently, a release of zinc from metallothionein mediated by interactions with GSH and the oxidized form of glutathione through S-thiolation has been reported.^34^,^35^ Evidence suggests that zinc released from metallothionein is facilitated by a directly coupled interaction of GSH and the oxidized form of glutathione with metallothionein (301) via the thiolate ligands that confer redox activity on zinc clusters. This results in an oxido reductive metallothionein-Zn complex.^36^ This finding confirms the results of our study, demonstrating the presence of a negative correlation coefficient between metallothionein, glutathione, and catalase values (*Figures 2 and 3*). Metallothionein may be acting as a sensor of the localized intracellular redox balance and may itself influence redox balance through GSH and the known antioxidant properties of zinc. Hartwig suggested that “the control of cellular zinc distribution as a function of the energy state of the cell is the long sought role of metallothionein”.^37^

Metallothionein is thought to play a major role in metal detoxification, supported by extensive evidence from both in vivo and in vitro studies. After exposure to various metals, there is a significant increase of metallothionein in tissues of the kidney, liver and intestine. Likewise, various cell types have been shown to accumulate metallothionein after metal exposure. Some metals such as lead are known to induce and bind to other intracellular proteins, which may also play a role in their detoxification.^34^

Glutathione has also been implicated in metal detoxification. Several in vivo and in vitro studies have reported increased sensitivities towards the toxic effects of mercury and cadmium following a depletion in GSH levels. Glutathione, however, appears tobe the first line of defense against cadmium toxicity preceding metallothionein induction.^34^

A number of metallothionein inducers, such as glucocorticoids, lipopolysaccharides, steroid hormones, cytokines and tumor necrosis factors, among others, can influence apoptosis in certain cells. This and other experimental data would seem to suggest that metallothionein plays a role in the apoptotic process.^36^

The results of the present study found that the fishermen group was exposed to various types of heavy metals as evidenced by the presence of cadmium, chromium, copper, lead and zinc in their blood, generating severe oxidative stress as manifested by the presence of high levels of malondialdehyde, accompanied by a severe decrease in blood antioxidant defense (decrease in glutathione content and catalase enzyme activity).

The induction of oxidative stress is an attractive hypothesis to explain the mutagenic and carcinogenic effects of metals. Ions of the carcinogenic metals, such as antimony, arsenic, chromium, cobalt, nickel and vanadium, are capable of performing redox reactions in biological systems. They have been shown to induce reactive oxygen and nitrogen species in vivo and in vitro in mammalian cells. The formation of hydroxyl radicals, most likely by Fenton and Haber-Weiss type reactions, have been detected.

These radicals are known to cause oxidative damage to lipids, proteins and DNA.^37^

Although the ions of the carcinogenic metal cadmium are not capable of exerting redox reactions in biological systems, they have been found to generate oxidative stress. The mechanism behind this property of cadmium seems to be the inhibition of antioxidative enzymes in vitro and in vivo. Cadmium has been shown to inhibit catalase, superoxide dismutase, glutathione reductase, and glutathione peroxidase.^37^

Finally, metal compounds exerting no redox chemistry such as cadmium may also contribute to elevated levels of oxidatively damaged DNA, which may be attributed to an inhibition of ROS-detoxifying enzymes such as superoxide dismutase.^37–39^ An inhibition of the DNA repair systems involved in the removal of oxidatively generated

DNA lesions may increase their steady state levels upon chronic exposure to metal compounds. Because these inhibitions have frequently been observed at far lower concentrations than with the induction of considerable amounts of oxidatively generated DNA lesions, as in the case of nickel and cadmium compounds, they may be particularly relevant to metal-induced carcinogenicity.^39^

## Conclusion

The present study found that the El Maadiya region is polluted with heavy metals, inducing oxidative stress in fishermen in the vicinity. The risk persists due to a large increase in the levels of malondialdehyde coinciding with a large decrease in the levels of the antioxidant glutathione and the enzymatic activities of catalase. The current results demonstrate the presence of highly significant levels of metallothioneinin the blood of local fishermen along with a positive correlation between metallothionein and malondialdehyde and a negative correlation between metallothionein and the antioxidant glutathione. These results reveal the necessity of further environmental monitoring in the study area in order to evaluate other types of pollutants and their effects on human health.

## References

[1] Alam MG, Tanaka A, Allinson G, Laurenson LJ, Stagnitti F, Snow ET. A comparison of trace element concentrations in cultured and wild carp (Cyprinuscarpio) of Lake Kasumigaura, Japan. *Ecotoxicol Environ Saf* [Internet] 2002 11 [cited 2016 Nov 22]; 53 3: 348– 54. Available from: http://www.sciencedirect.com/science/article/pii/S014765130200012X Subscription required to view. 12485577 10.1016/s0147-6513(02)00012-x

[2] El Nemr A, Khaled A, Moneer AA, El Sikaily A. Risk probability due to heavy metals in bivalve from Egyptian Mediterranean coast. *Egypt J Aquat Res* [Internet] 2012 [cited 2016 Nov 22]; 38 2: 67– 75. Available from: http://www.sciencedirect.com/science/article/pii/S1687428512000106 Subscription required to view.

[3] Canli M, Atli G The relationships between heavy metal (Cd, Cr, Cu, Fe, Pb, Zn) levels and the size of six Mediterranean fish species. Environ Pollut [Internet] 2003 [cited 2016 Nov 22]; 121 1: 129– 36. Available from: https://www.researchgate.net/publication/10997179_The_relationship_between_heavy_metal_Cd_Cr_Cu_Fe_Pb_Zn_levels_and_the_size_of_six_Mediterranean_fish_species 12475070 10.1016/s0269-7491(02)00194-x

[4] Sun J, Rong J, Zheng Y, Ma D, Lan X. Risk assessment of heavy metal contaminated Dagu River sediments. *Procedia Environ Sci* [Internet] 2011 [cited 2016 Nov 22]; 8: 764– 72. Available from: http://www.sciencedirect.com/science/article/pii/S1878029611007523

[5] El Nemr AM, El Sikaily A, Khaled A. Total and leachable heavy metals in muddy and sandy sediments of Egyptian coast along Mediterranean Sea. *Environ Monit Assess* [Internet] 2007 6 [cited 2016 Nov 22]; 129 1–3: 151– 68. Available from: http://link.springer.com/article/10.1007%2Fs10661-006-9349-8 Subscription required to view. 17057978 10.1007/s10661-006-9349-8

[6] El-Sikaily A, Khaled A, El-Nemr A. Heavy metals monitoring using bivalves from Mediterranean Sea and Red Sea. *Environ Monit Assess* [Internet] 2004 11 [cited 2016 Nov 23]; 98 1–3: 41– 58. Available from: http://link.springer.com/article/10.1023/B:EMAS.0000038178.98985.5d Subscription required to view. 15473528 10.1023/b:emas.0000038178.98985.5d

[7] Viarengo A, Burlando B, Dondero F, Marro A, Fabbri R. Metallothionein as a tool in biomonitoring programmes. *Biomarkers* [Internet] 1999 [cited 2016 Nov 23]; 4 6: 455– 66. Available from: http://www.tandfonline.com/doi/abs/10.1080/135475099230615 Subscription required to view. 23902390 10.1080/135475099230615

[8] Yang M. A current global view of environmental and occupational cancers. *J Environ Sci Health C Environ Carcinog Ecotoxicol Rev* [Internet] 2011 7 [cited 2016 Nov 23]; 29 3: 223– 49. Available from: http://thirdworld.nl/a-current-global-view-of-environmental-and-occupational-cancers 21929381 10.1080/10590501.2011.601848

[9] Wise SS, Wise JP Aneuploidy as an early mechanistic event in metal carcinogenesis. *Biochem Soc Trans* [Internet] 2010 12 [cited 2016 Nov 23]; 38 6: 1650– 4. Available from: http://www.biochemsoctrans.org/content/38/6/1650.long 21118142 10.1042/BST0381650 PMC4086856

[10] Lee JC, Son YO, Pratheeshkumar P, Shi X. Oxidative stress and metal carcinogenesis. Free Radic Biol Med [Internet] 2012 8 15 [cited 2016 Nov 23]; 53 4: 742– 57. Available from: http://linkinghub.elsevier.com/retrieve/pii/S0891-5849(12)00337-1 Subscription required to view. 22705365 10.1016/j.freeradbiomed.2012.06.002

[11] Koedrith P, Seo YR Advances in carcinogenic metal toxicity and potential molecular markers. *Int J Mol Sci* [Internet] 2011 [cited 2016 Nov 23]; 12 12: 9576– 95. Available from: https://www.ncbi.nlm.nih.gov/pmc/articles/PMC3257147/ 22272150 10.3390/ijms12129576 PMC3257147

[12] Koedrith P, Kim H, Weon JI, Seo YR. Toxicogenomic approaches for understanding molecular mechanisms of heavy metal mutagenicity and carcinogenicity. *Int J Hyg Environ Health* [Internet] 2013 8 [cited 2016 Nov 23]; 216 5: 587– 98. Available from: http://www.sciencedirect.com/science/article/pii/S1438463913000370 Subscription required to view. 23540489 10.1016/j.ijheh.2013.02.010

[13] Dahab OA. Chromium biogeochemical cycle in Abu Kir Bay, east of Alexandria, Egypt. *Estuar Coastal Shelf Sci* [Internet] 1989 10 [cited 2016 Nov 23]; 29 4: 327– 40. Available from: http://www.sciencedirect.com/science/article/pii/0272771489900322 Subscription required to view.

[14] Viarengo A, Ponzano E, Dondero F, Fabbri R. A simple spectrophotometric method for metallothionein evaluation in marine organisms: an application to Mediterranean and Antarctic mollusks. *Marine Environ Res* [Internet] 1997 7 [cited 2016 Nov 23]; 44 1: 69– 84. Available from: http://www.sciencedirect.com/science/article/pii/S0141113696001031 Subscription required to view.

[15] Christensen JM, Poulsen OM, Anglov T. Protocol for the design and interpretation of method evaluation in atomic absorption spectrometric analysis. Application to the determination of lead and manganese in blood. *J Anal Atomic Spectrom* [Internet] 1992 3 [cited 2016 Nov 23]; 7 2: 329– 34. Available from: http://pubs.rsc.org/en/Content/ArticleLanding/1992/JA/ja9920700329#!divAbstract Subscription required to view.

[16] Grider A, Bailey LB, Cousins RJ. Erythrocyte metallothionein as an index of zinc status in humans. *Proc Natl Acad Sci USA* [Internet] 1990 2 [cited 2016 Nov 23]; 87 4: 1259– 62. Available from: http://www.pnas.org/content/87/4/1259.full.pdf 2304897 10.1073/pnas.87.4.1259 PMC53453

[17] Zeneli L, Daci NH, Daci-Ajvazi MN, Pacarizi H. Effects of pollution on lead and cadmium concentration and correlation with biochemical parameters in blood of human population nearby Kosovo thermo power plants. *Am J Biochem Biotechnol* [Internet] 2008 [cited 2016 Nov 23]; 4 3: 273– 6. Available from: http://thescipub.com/PDF/ajbbsp.2008.273.276.pdf

[18] Draper HH, Hadley M Malondialdehyde determination as index of lipid peroxidation. *Methods Enzymol* [Internet] 1990 [cited 2016 Nov 23]; 186: 421– 31. Available from: http://www.sciencedirect.com/science/article/pii/007668799086135I Subscription required to view. 2233309 10.1016/0076-6879(90)86135-i

[19] Beutler E, Duron O, Kelly BM. Improved method for the determination of blood glutathione. *J Lab Clin Med*. 1963; 61: 882– 8. 13967893

[20] Donald W, Hugo E Enzymes I: oxidoreductases, transferases. : Bergmeyer HU, Bergmeyer J, Grassi M, Methods of enzymatic analysis. 3rd ed Weinheim, Germany: Wiley-VCH; 1987 p 273– 86.

[21] El Nemr A. Assessment of heavy metal pollution in surface muddy sediments of Lake Burullus, southeastern Mediterranean, Egypt. *Egypt J Aquat Biol Fish* [Internet] 2003 10 [cited 2016 Nov 23]; 7 4: 67– 90. Available from: https://www.researchgate.net/publication/235224071_Assessment_of_heavy_metal_pollution_in_surface_muddy_sediments_of_Lake_Burullus_southeastern_Mediterranean_Egypt

[22] Geret F, Cosson RF Induction of specific isoforms of metallothionein in mussel tissues after exposure to cadmium or mercury. *Arch Environ Contam Toxicol* [Internet] 2002 [cited 2016 Nov 23]; 42: 36– 42. Available from: http://www.academia.edu/17511356/Induction_of_Specific_Isoforms_of_Metallothionein_in_Mussel_Tissues_After_Exposure_to_Cadmium_or_Mercury 11706366 10.1007/s002440010289

[23] Amiard JC, Amiard-Triquet C, Barka S, Pellerin J, Rainbow PS. Metallothioneins in aquatic invertebrates: their role in metal detoxification and their use as biomarkers. *Aquat Toxicol* [Internet] 2006 2 10 [cited 2016 Nov 23]; 76 2: 160– 202. Available from: http://www.sciencedirect.com/science/article/pii/S0166445X05003279 Subscription required to view. 16289342 10.1016/j.aquatox.2005.08.015

[24] Perceval O, Couillard Y, Pinel-Alloul B, Bonneris E, Campbell PG. Long-term trends in accumulated metals (Cd, Cu and Zn) and metallothionein in bivalves from lakes within a smelter-impacted region. *Sci Total Environ* [Internet] 2006 10 1 [cited 2016 Nov 23]; 369 1–3: 403– 18. Available from: http://linkinghub.elsevier.com/retrieve/pii/S0048-9697(06)00312-3 Subscription required to view. 16814847 10.1016/j.scitotenv.2006.04.019

[25] Monserrat JM, Martienez PE, Geracitano LA, Amado LL, Martins CM, Pinho GL, Chaves IS, Ferreira-Cravo M, Ventura-Lima J, Bianchini A. Pollution biomarkers in estuarine animals: critical review and new perspectives. Comp BiochemPhysiol Part C: Toxico Pharmacol [Internet] 2007 Jul-Aug [cited 2016 Nov 23]; 146 1–2: 221– 34. Available from: http://www.sciencedirect.com/science/article/pii/S1532045606001992 Subscription required to view. 10.1016/j.cbpc.2006.08.012 17045848

[26] Machreki-Ajmi M, Hamza-Chaffai A Assessment of sediment/water contamination by in vivo transplantation of the cockles Cerastodermaglaucum from a non contaminated to a contaminated area by cadmium. *Ecotoxicol* [Internet] 2008 11 [cited 2016 Nov 23]; 17 8: 802– 10. Available from: http://link.springer.com/article/10.1007/s10646-008-0238-5 Subscription required to view. 10.1007/s10646-008-0238-5 18574691

[27] Paul-Pont I, de Montaudouin X, Gonzalez P, Soudant P, Baudrimont M. How life history contributes to stress response in the Manila clam Ruditapesphilippinarum. *Environ Sci Pollut Res* [Internet] 2010 5 [cited 2016 Nov 23]; 17 4: 987– 98. Available from: http://link.springer.com/article/10.1007/s11356-009-0283-5 Subscription required to view. 10.1007/s11356-009-0283-5 20099041

[28] Haq F, Mahoney M, Koropatnick J. Signaling events for metallothionein induction. *Mutat Res* [Internet] 2003 12 10 [cited 2016 Nov 23]; 533 1–2: 211– 26. Available from: http://www.sciencedirect.com/science/article/pii/S0027510703002185 Subscription required to view. 14643422 10.1016/j.mrfmmm.2003.07.014

[29] Carpene E, Andreani G, Isani G. Metallothionein functions and structural characteristics. *J Trace Elem Med Biol* [Internet] 2007 12 11 [cited 2016 Nov 23]; 21Suppl 1: 35– 9. Available from: http://linkinghub.elsevier.com/retrieve/pii/S0946-672X(07)00107-1 Subscription required to view. 10.1016/j.jtemb.2007.09.011 18039494

[30] Simpkins CO. Metallothionein in human disease. *Cell Mol Biol* (Noisy-le-grand) [Internet] 2000 3 [cited 2016 Nov 23]; 46 2: 465– 88. Available from: https://www.researchgate.net/publication/12541671_Metallothionein_in_human_disease Subscription required to view. 10774934

[31] Lynes MA, Fontenot AP, Lawrence DA, Rosenspire AJ, Pollard KM. Gene expression influences on metal immunomodulation. *Toxicol Appl Pharmacol* [Internet] 2006 1 1 [cited 2016 Nov 23]; 210 1–2: 9– 16. Available from: http://linkinghub.elsevier.com/retrieve/pii/S0041-008X(05)00210-3 Subscription required to view. 15993910 10.1016/j.taap.2005.04.021

[32] Crowthers KC, Kline V, Giardina C, Lynes MA. Augmented humoral immune function in metallothionein-null mice. *Toxicol Appl Pharmacol* [Internet] 2000 8 1 [cited 2016 Nov 23]; 166 3: 161– 72. Available from: http://www.sciencedirect.com/science/article/pii/S0041008X00989610 Subscription required to view. 10906280 10.1006/taap.2000.8961

[33] Kim CH, Kim JH, Lee J, Ahn YS. Zinc-induced NF-kappaB inhibition can be modulated by changes in the intracellular metallothionein level. *Toxicol Appl Pharmacol* [Internet] 2003 7 15 [cited 2016 Nov 23]; 190 2: 189– 96. Available from: http://www.sciencedirect.com/science/article/pii/S0041008X03001674 Subscription required to view. 12878048 10.1016/s0041-008x(03)00167-4

[34] Miles AT, Hawksworth GM, Beattie JH, Rodilla V. Induction, regulation, degradation, and biological significance of mammalian metallothioneins. *Crit Rev Biochem Mol Biol* [Internet] 2000 [cited 2016 Nov 23]; 35 1: 35– 70. Available from: http://www.tandfonline.com/doi/abs/10.1080/10409230091169168 Subscription required to view. 10755665 10.1080/10409230091169168

[35] Maret W. Zinc coordination environments in proteins as redox sensors and signal transducers. *Antioxid Redox Signal* [Internet] 2006 Sep-Oct [cited 2016 Nov 23]; 8 9–10: 1419– 41. Available from: http://online.liebertpub.com/doi/abs/10.1089/ars.2006.8.1419 Subscription required to view. 16987000 10.1089/ars.2006.8.1419

[36] Formigari A, Irato P, Santon A. Zinc, antioxidant systems and metallothionein in metal mediated-apoptosis: biochemical and cytochemical aspects. *Comp Biochem Physiol C Toxicol Pharmacol* [Internet] 2007 11 [cited 2016 Nov 23]; 146 4: 443– 59. Available from: https://www.researchgate.net/publication/6122984_Zinc_antioxidant_systems_and_metallothionein_in_metal_mediated-apoptosis_Biochemical_and_cytochemical_aspects 17716951 10.1016/j.cbpc.2007.07.010

[37] Hartwig A. Metal interaction with redox regulation: an integrating concept in metal carcinogenesis? *Free Radic Biol Med* [Internet] 2013 2 [cited 2016 Nov 23]; 55: 63– 72. Available from: http://linkinghub.elsevier.com/retrieve/pii/S0891-5849(12)01818-7 23183323 10.1016/j.freeradbiomed.2012.11.009

[38] Valko M, Rhodes CJ, Moncol J, Izakovic M, Mazur M. Free radicals, metals and antioxidants in oxidative stress-induced cancer. *Chem Biol Interact* [Internet] 2006 3 10 [cited 2016 Nov 23]; 160 1: 1– 40. Available from: http://www.sciencedirect.com/science/article/pii/S0009279705004333 Subscription required to view. 16430879 10.1016/j.cbi.2005.12.009

[39] Salnikow K, Zhitkovich A Genetic and epigenetic mechanisms in metal carcinogenesis and cocarcinogenesis: nickel, arsenic, and chromium. *Chem Res Toxicol* [Internet] 2008 1 [cited 2016 Nov 23]; 21 1: 28– 44. Available from: http://pubs.acs.org/doi/abs/10.1021/tx700198a 17970581 10.1021/tx700198a PMC2602826

